# Study on follicular fluid metabolomics components at different ages based on lipid metabolism

**DOI:** 10.1186/s12958-020-00599-8

**Published:** 2020-05-12

**Authors:** Xingxing Zhang, Tianqi Wang, Jingyan Song, Jifeng Deng, Zhengao Sun

**Affiliations:** 1 Maternity and Child Health Care of ZaoZhuang, ZaoZhuang, 277100 Shandong China; 2 Traditional Chinese Medicine History and Literature, Institute for Literature and Culture of Chinese Medicine, Shandong University of Traditional Chinese Medicine, Jinan, 250355 China; 3 Department of Gynecology and Obstetrics of Traditional Chinese Medicine, The First Clinical College, Shandong University of Traditional Chinese Medicine, Jinan, 250014 China; 4 School of Bioscience & Bioengineering, South China University of Technology, Guangzhou, 510640 China; 5 Reproductive and Genetic Center of Integrated Traditional and Western Medicine, The Affiliated Hospital of Shandong University of Traditional Chinese Medicine, Jinan, 250011 China

**Keywords:** Age, Oocytes, Lipid metabolism, Follicular fluid, Metabolomics

## Abstract

**Background:**

Follicular fluid is an important external environment for the growth and development of oocytes. A thorough identification of specific components in follicular fluid can better the existing understand of intracellular signal transduction and reveal potential biomarkers of oocyte health in women undergoing assisted reproductive therapy. To study on follicular fluid metabolomics components at different ages based on lipid metabolism, we have adopted a new method of SWATH to MRM(the sequential window acquisition of all theoretical fragment-ion spectra to multiple reaction monitor)metabolomics to provide extensive coverage and excellent quantitative data. This was done to investigate the differences in follicular fluid of patients undergoing in vitro fertilization (IVF) and embryo transfer in different age groups and to further explore the relationship between follicular fluid, age and reproductive function.

**Method:**

A combination of Ultra-high-performance liquid chromatography and high resolution mass spectrometry techniques were used to analyze the follicular fluid of 230 patients enrolled for the IVF cycle. The patients were of different ages grouped into two groups:the younger and older patients.The obtained multidimensional chromatographic data were processed by principal component analysis (PCA) and partial least squares discriminant analysis (PLS-DA). The charge ratios and mass numbers enabled for the identification of different fragments in the samples. Matching information obtained through database search and the fragment information obtained by fragment ion scan structurally identified substances in the samples. This was used to determine the differential compounds.

**Results:**

The quality of oocytes decline with age,and the lipid composition in follicular fluid also changes,The lipid metabolism that changes with age may be related to the quality of oocytes.The main differences were in lipid metabolites. Some were up-regulated: Arachidonate, LysoPC(16:1), LysoPC(20:4) and LysoPC(20:3) while others were down-regulated: LysoPC(18:3) and LysoPC(18:1).

**Conclusions:**

Metabolomic analysis of follicular fluid revealed that with the increase in age, several differential metabolites are at play. Among these metabolites, lipid metabolism undergoes significant changes that affect the development of oocytes thus causing reduced fertility in older women. These differential metabolites related to follicular development may provide possible detection and treatment targets for promoting oocyte health, and provide scientific basis for understanding the environment of oocyte development.

## Introduction

Infertility is a worldwide problem affecting couples in their childbearing age. It is one of the three major diseases affecting human life and health. Development of social economy has led to a change in the concept of marriage and childbearing. The reproductive age of women has gone higher, and more older women are having pregnancy requirements. However, the reproductive capability of more older women is declining [1]. Finding solutions to fertility problems of aging women has become of paramount importance in reproductive medicine.However,the mechanism of age-related decline in reproductive function is still unclear. Numerous studies have shown that its mechanism may be related to a series of changes such as increased oocyte apoptosis, oxidative stress disorder, energy conversion disorder, and endocrine and metabolic abnormalities caused by age [2–6].

Follicular fluid is the culture medium in which an oocyte grows and differentiates. It directly or indirectly influences the fertility and up growth potency of oocytes thus indirectly affecting women’s reproductive function.Changes in follicular microenvironment in older women may be responsible for altered protein function thus affecting the viability of oocytes. This undoubtedly causes adverse pregnancy outcomes [7, 8].

Metabolomics is an emerging omics technology developed after genomics and proteomics.It enables simultaneous qualitative and quantitative analysis of all metabolites of an organ, tissue or cells in a specific physiological period or condition in order to find target differential metabolites. It can be used for early diagnosis of disease, drug target discovery and disease mechanism research. Rapid development of metabolomics have enabled the use of follicular fluid as an objective evaluation index to reflect the quality of oocytes. This plays an important role in predicting the outcome of an in-vitro fertilization (IVF) cycle. Researchers in reproductive medicine have identified follicular fluid as a biomarker source for evaluating oocyte quality. Changes in its composition affect oocyte quality.Results obtained from the metabolomics analysis of follicular fluid have become an important indicator of clinical prediction of IVF outcomes [9].Previous studies have shown that the composition of the follicular fluid changes with age, which may be an important cause of the aging of oocytes [10]. These studies also revealed that the change process of female reproductive function may be the result of comprehensive regulation of multiple aspects and approaches. This study used metabolomics to analyze human follicular fluids (HFF) from patients of different ages.It opened up a new direction for exploring the mechanism of oocyte aging and provided new ideas and methods for treating senile infertility as well as improving IVF cycle outcomes.

## Materials and methods

### Experimental chemicals

The internal standards, including isotope-labeled Arachidonate (AA), isotope-labeled 16:1(d7) lysophosphatidylcholine(LPC),isotope-labeled 20:4(d7) LPC,isotope-labeled 20:3 (d7) LPC, isotope-labeled 18:3 (d7) LPC and Isotope-labeled 18:1 (d7) LPC, were purchased from Sigma-Aldrich (St. Louis, MO, USA). Distilled water was obtained from a Milli-Q system (Millipore, MA, USA). Chromatographic grade formic acid, acetonitrile and methanol were purchased from Fisher (Fairlawn, NJ, USA).

### Sample collection and preparation

Source of cases: 230 cases of patients undergoing IVF-ET between August, 2016 and June, 2017, at the center of integrated traditional Chinese and western medicine reproductive and genetic center of affiliated hospital of Shandong university of traditional Chinese medicine. The study was approved by the Health Authorities and Ethics Committees of Shandong University of Traditional Chinese Medicine Affiliated Hospital. Diagnostic criteria: Previous pregnancy history. No contraception use for 12 months after pregnancy was regarded as secondary infertility [11].Inclusion criteria: Patients aged between 21 and 48 years meeting the diagnostic criteria of infertility caused by simple tubal factors. They also had to have no major gynecological diseases or other major diseases, have a body mass index (BMI) ranging between 18.5 and 24.9 kg/m^2^ [12] and sign an informed consent.Exclusion criteria: Patients with infertility due to other factors such ascongenital ovarian dysplasia, serious malformation of reproductive organs, those with a major operation history, those with infertility due to male factors, those that have used hormone drugs within 3 months prior to the study. Grouping: Patients who met the inclusion criteria were first divided into two groups: a younger group (140 cases) of those aged between 28 and 34 years, and and an older group (90 cases) of those between 35 and 48 years. (World health care (WHO) and international association for obstetrics and gynecology scientific regulations). After identifying the major differences between the two groups, the 230 cases were reclassified into four groups: group A (54 cases) of those aged between 21 and 27 years old, group B (85 cases) of those aged between 28 and 34 years old, group C (51 cases) of those aged between 35 and 41 years old, and group D (40 cases) of those aged between 42 and 48 years old. This was done to observe the specific changes in follicular fluids between the four groups.

All subjects underwent controlled ovarian hyperstimulation according to our established protocols.All patients were enrolled in the controlled super-stimulatory antagonist program (GnRH-ant program).In the program, patients were first tested for serum sex hormone levels, ultrasonography was then done on the third day of their menstrual cycle to assess ovarian function. They then received recombinant follicle stimulating hormone (r-FSH, cognac) or/and injection with human gonadotropin (HMG) at astarting dose of 300 IU per day for 4 days. After 4 days of follow-up, an ultrasound examination to monitor follicular development was done and adjustments of the r-FSH, cognac/HMG dosage made. Once the dominant follicle had a diameter of 13 to 14 mm, 0.25 mg of leuprorelin Acetate per day was administered through injection. This was done until the day the injection with human chorionic gonadotropin (HCG) / recombinant-human chorionic gonadotropin (r-HCG) was administered.

Follicular fluid collection: After the laboratory licked the eggs leave the first tube of FF place it in the original test tube let it stand for 10 min at room temperature take all the supernatant and place it in a 15 ml centrifuge tube for centrifugation The constant rotation speed is 2000 r/min the centrifugation time is 5 min and the supernatant is taken up to 1.5 ml EP tube and stored in a − 80 °C ultra-low temperature storage box for experiment and avoid repeated freezing or thawing.

### Serum hormone measurement and follicle calculation

Circulating levels of hormones, including serum FSH, LH, E2, testosterone (detected at day 2), E2 and P (detected at hCG day), were measured using a radioimmunoassay method. The numbers of antral follicles were counted using ultrasonography on day 2.

### Measurement of the reproducibility and accuracy of SWATH mass spectrometry

Before analysis, eleven follicular fluid samples were thawed at room temperature for quality control (QC) (one QC after every four follicular fluid samples). First, samples were prepared by mixing 100 μL of each individual follicular fluid samples. To reduce the effect of the solvent and obtain a good peak shape, a total volume of 150 μL of follicular fluid or QC sample was mixed with 450 μL of methanol (v/v, 1:3) containing isotope-labeled Arachidonate(AA),isotope-labeled 16:1(d7) lysophosphatidylcholine(LPC),isotope-labeled 20:4(d7) LPC,isotope-labeled 20:3 (d7) LPC, isotope-labeled 18:3 (d7) LPC and Isotope-labeled 18:1 (d7) LPC. Next, the mixture was vortexed for 10 min and centrifuged at 13000×g for 20 min at 15 °C.The contents of the supernatant were analyzed using UPLC-Q-TOF.

### Method conditions

Aliquots of 5 μL of the supernatant were injected into the UPLC tandem Triple TOF 5600 system (SCIEX, CA, USA) in random order. A reverse-phase 2.1*100 mm ACQUITY 1.7 μm C18 column (Waters, Ireland) was used for separation. A gradient mobile phase composed of 0.05% formic acid solution (A) and acetonitrile (B) was used and kept at 90% A for 0.5 min, increased to 95% B over the next 6.5 min, and then returned to 90% A from 8.5 min to 8.6 min. The total running time was 13 min. The mass parameters were as follows: nebulizing gas, 55 psi; TIS gas, 55 psi; source temperature, 500 °C; and ion spray voltage, 5000 V with 35 psi curtain gas in positive mode and − 4000 V with 35 psi curtain gas in negative mode. The declustering potential and collision energy were set at 55 V and 40 ± 20 V, respectively, in positive mode (− 55 V and − 40 ± 20 V, respectively, in negative mode). The SWATH method with 20 variable isolation windows was performed in TOF MS full-scan mode and in TOF MS/MS product ion scan mode at m/ z 50–1200 in Analyst TF 1.7.1 software.

### Data collection, processing and statistical analysis

Analysis was done using SPSS 22.0 statistical software. *P* value of less than 0.05 (*P* < 0.05) was considered statistically significant. The quantitative data of each group was normally distributed and hence the means and standard deviation was used for statistical description. The data was not statistically described using the median and quartile deviation. The changes in values before and after treatment in the two groups (the younger and the older) were compared with the normal distribution using the independent sample t test, and the rank sum test without the normal distribution was compared. The count data of the two groups were statistically described using their frequency (composition ratio)and the chi-square test.

The multi-dimensional chromatographic data obtained were transformed into a matrix using metabolomic analysis methods: partial least squares-discriminant analysis (PCA and PLS-DA). In the scoring plot, each point represented a corresponding sample while in the loading plot, discrete points represented the variables separated in the score plot. Those with higher dispersion appeared more in the score plot. The *P* value for each variable was determined using the t test method. A variable with a *P* value of more than 0.01 and less than 0.05,0.01 < *P* < 0.05 was said to be significant while those with a *P* value of less than 0.01,*P* < 0.01was said to be very significant. According to the primary and secondary mass spectrum information of the groups’ metabolic pathways, there were differences in charge ratio, mass number and isotope abundance.

## Results

### Characteristics of patients

From Table 1, there were significant differences (*P* < 0.01) in age, basic follicle stimulating hormone (bFSH), basic luteinizing hormone (bLH) and basic estradiol (bE_2_) between the younger and the older group. The basic antral follicle count (bAFC) in the older group was significantly lower (*P* < 0.05) compared to that in the younger group This was an indication that the ovarian function of the older group was significantly low. However, there was no significant difference in Body Mass Index (BMI) between the two groups (*P* > 0.05).

**Table 1:** Between-group comparison of demographic and clinical characteristics

Item	Younger group	Older group	*P*-value
Age (year)	29.38 ± 3.01	40.51 ± 3.47	< 0.001*
BMI (kg/m^2^)	21.62 ± 6.67	22.79 ± 1.34	0.05
bFSH (IU/L)	7.49 ± 1.88	9.32 ± 6.12	< 0.001*
bLH (IU/L)	5.04 ± 3.07	5.70 ± 6.70	0.001*
bE_2_ (pg/ml)	44.95 ± 22.11	50.66 ± 36.30	< 0.001*
bAFC (n)	15.65 ± 5.83	10.46 ± 5.91	0.001*

*Significant at *P* < 0.05

### Ovulation outcomes

Oocytes retrieval rate, 2PN cleavage rate and transferable embryo rate decreased with age. In all the difference between the younger and older group was statistically significant with *P* values of < 0.001, 0.002 and < 0.001 respectively. However, there was no significant difference (*P* = 0.077) in High-quality embryo rate between the younger age group and the older age group (Table 2).

**Table 2:** Between-group comparison of ovulation outcomes

Item	Younger group	Older group	χ^2^	*P*-value
Oocytes retrieved rate (%)	88.0	66.5	162.8	< 0.001*
2PN fertilization rate (%)	88.3	77.1	9.8	0.002*
2PN cleavage rate (%)	98.0	84.3	51.3	< 0.001*
Transferable embryo rate (%)	72.8	55.7	39.1	< 0.001*
High-quality embryo rate (%)	45.0	38.2	3.25	0.077

*Significant at *P* < 0.05

Note: Transferable embryo rate = number of transferable embryos/2PN cleavage number; High-quality embryo rate = number of high-quality embryos/2PN cleavage number

### Significantly significant substance in follicular fluid between younger and older groups

It was found that there were Fourteen (14) different compounds in the two groups, 8 of which involved lipid metabolism as shown in Table 3.

**Table 3:** Significant differences in follicular fluid between younger and older groups

Compound	Molecular Formula	Molecular weight	Charge-mass ratio	Pathway
4,5-Dihydroorotic acid	C_5_H_6_N_2_O_4_	158.0327	159.0434	Pyrimidine metabolism
Maleylacetoacetic acid	C_8_H_8_O_6_	200.0321	201.0591	Aromatic nucleus metabolism
4-oxo-Retinoic acid	C_20_H_26_O_3_	314.1882	315.1931	Vitamin metabolism
Arachidonate	C_20_H_32_O_2_	303.2321	304.2243	Lipid metabolism
Nicotine glucuronide	C_16_H_22_N_2_O_6_	338.1478	339.1428	Glucose metabolism
DG(14:1(9Z)/22:2(13Z,16Z)/0:0)	C_39_H_70_O_5_	618.5223	619.5407	Lipid metabolism
TG(18:1(11Z)/24:0/20:5(5Z,8Z,11Z,14Z,17Z))	C_65_H_114_O_6_	990.8615	Only in old woman	Lipid metabolism
Deoxycorticosterone	C_21_H_30_O_3_	330.2195	331.2139	Biosynthesis of mineralocorticoid
LysoPC(14:0)	C_22_H_46_NO_7_P	467.3012	468.3111	Lipid metabolism
LysoPC(16:1)	C_24_H_48_NO_7_P	495.3376	494.3222	Lipid metabolism
LysoPC(18:0)	C_26_H_54_NO_7_P	523.3638	524.3725	Lipid metabolism
Phytosphingosine	C_18_H_39_NO_3_	317.293	318.3007	Lipid metabolism
Phosphaticlylcholine(16:0/22:0)	C_46_H_94_NO_7_P	323.1236	Only in old woman	Lipid metabolism
5,6-Dihydrouridine	C_9_H_14_N_2_O_6_	246.0852	247.074	Nucleic acid metabolism

**Fig. 1:**
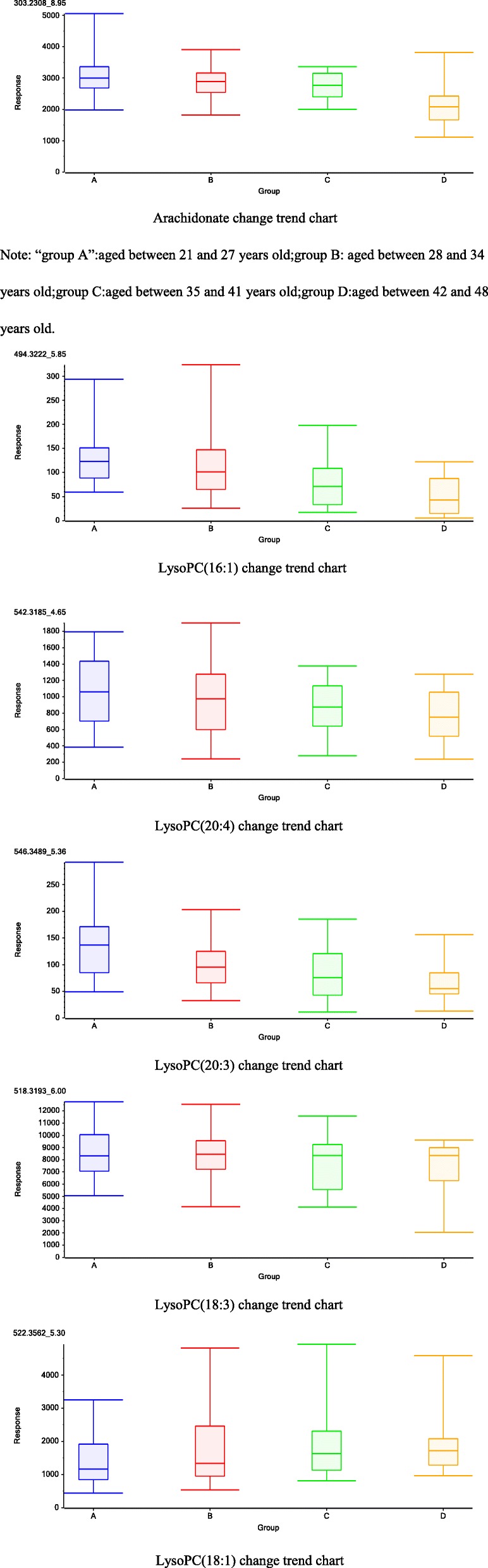
Arachidonate change trend chart. Note: “group A”:aged between 21 and 27 years old;group B: aged between 28 and 34 years old;group C:aged between 35 and 41 years old;group D:aged between 42 and 48 years old. LysoPC(16:1) change trend chart. LysoPC(20:4) change trend chart. LysoPC(20:3) change trend chart. LysoPC(18:3) change trend chart. LysoPC(18:1) change trend chart

To investigate the changes in lipid specific metabolites with age, we divided the patients into four groups based on age and analyzed their follicular fluid again. Six substances that were differentially regulated were detected: Arachidonate (AA), LysoPC(16:1), LysoPC(20:4), LysoPC(20:3), LysoPC(18:3) and LysoPC(18:1). The quantitative map results showed that there were four down-regulated substances:Arachidonate, LysoPC(16:1), LysoPC(20:4) and LysoPC(20:3) and two up-regulated substances: LysoPC(18:3) and LysoPC(18:1) (Table 4) (Fig. 1).

**Table 4:** the differential lipid metabolites in follicular fluid among the four groups

Compound	T_R_ (min)	m/z	Molecular Formula	Identity	MS/MS Fragments	T-test (p)	Pathway
1	8.9	303.2321	C_20_H_32_O_2_	Arachidonate↓	59.0102; 205.1184; 259.1355; 285.0981	< 0.01	Lipid metabolism
2	4.4	494.3222	C_24_H_48_NO_7_P	LysoPC(16:1) ↓	104.1066; 184.0726; 311.2562; 476.3114	< 0.01	Lipid metabolism
3	5.8	542.3185	C_28_H_50_NO_7_P	LysoPC(20:4) ↓	86.0989; 146.9819; 337.2730; 483.2480	< 0.01	Lipid metabolism
4	6.6	546.3489	C_28_H_52_NO_7_P	LysoPC(20:3) ↓	104.1081; 184.0714; 341.3021; 487.2795	< 0.01	Lipid metabolism
5	4.7	518.3193	C_26_H_48_NO_7_P	LysoPC(18:3) ↑	104.1072; 146.9842; 313.2700; 459.2479	< 0.01	Lipid metabolism
6	5.3	522.3562	C_26_H_52_NO_7_P	LysoPC(18:1) ↑	104.1058; 184.0724; 504.3413	< 0.01	Lipid metabolism

Note: “↓”: down-regulated with age; “↑”: up-regulated with age

## Discussions

In the present study, we differentiated the characteristics of metabolic ions in follicular fluid in the younger and older patients and identified several differential metabolites.

Metabonomic analysis of follicular fluid showed that several major metabolites changed with age, with some upregulated: arachidonic acid, LysoPC (16:1), LysoPC (20:4) and LysoPC (20:3), while others downregulated: LysoPC (18:3) and LysoPC (18:1). Among these metabolites, significant changes will take place in lipid metabolism, mainly characterized by the up-regulation of arachidonic acid, which affects oocyte development, while LPC participates in the regulation of follicular development and oocyte maturation, and its complex changes are closely related to follicular development. These differential metabolites related to follicular development may provide possible detection and therapeutic targets for promoting oocyte health. To provide scientific basis for understanding the environment of oocyte development.

Lipids is the general term used to refer to oils, fats, and lipids. They are important nutrients needed by the human body. They supply energy needed by the body as well as provide essential fatty acids for the body. Similarly, lipids are important energy support for oocyte growth and development; they participate in the construction of its membrane, regulate cell cycle and survival, malignant transformation and apoptosis [7–9].. It can be assumed that as age increases, lipid metabolism in the follicular fluid may cause a change in the oocyte. However,no study has shown an existing relationship between lipid metabolism oocyte aging. This study aimed to explore the role of lipid metabolism in oocyte aging in two different age groups through follicular fluid metabolomics It at the same time focused on opening up new directions for the treatment of senile infertility and provide new ideas and methods to improve IVF outcome. This study suggests that a decline in female fertility due to age might be related to the change in lipid metabolism in the follicular fluid.

Arachidonate (AA) is an omega-6 polyunsaturated fatty acid similar to the saturated peanut acid in peanut oil [1]. AA is an all-cis-5,8,11,14-eicosatetraenoic acid with a chemical formula: CH3(CH2)4(CH=CH-CH2)4(CH2)2COOH. It is a saturated fatty acid containing four carbon-carbon double bonds and one carbon-oxygen double bond. It is widely distributed in the animal kingdom though it is found in small amounts in a variety of glycerides and glycerophospholipids. It has been found that AA and niclosamide potentially induce apoptosis through the common pathway of cytochrome c release from mitochondria.This is consistent with the view that niclosamide partially mediates cell death through arachidonic acid. Interestingly, AA has been found to increase mitochondrial permeability transition (PT) and cytochrome c release [13].

In addition to the environment in which lipid metabolism affect follicular development, lipid peroxidation is also closely related to follicular development [14]. One study found that the expressions of arachidonic acid 12-lipoxygenase (Alox12) and arachidonic acid 15-lipoxygenase (Alox15) were significantly up-regulated in an obese diet reflecting an increase in lipid peroxidation. Lipid peroxidation is associated with many disease states associated with oxidative stress, aging and metabolic diseases [14]. Understanding of the multiple roles of lipoxygenases (other than in the classic arachidonic acid cascade) is increasing [15], creating a clearer picture of their role in generating the cellular redox balance.Lipoxygenases are pro-oxidative enzymes that by forming hydroperoxy lipids,can alter the redox state and gene expression pattern within the cell [16]. Apart from the implications for cellular redox balance, an increase in lipoxygenase activity also leads to greater oxidation of membrane lipids which in turn impairs the normal functions of the cell membrane and membrane-bound enzymes [17].Such impairments at the tissue level early in reproductive life may lead to increasing dysregulation of normal ovarian function later in life. Furthermore, there may be direct effects of *Alox12* expression on both ovulation and follicular reserve. As an example, *Alox12* is expressed in granulosa cells, thecal cells and follicular fluid at the time of ovulation in rats [18, 19],while inhibition of lipoxygenase can impair ovulation [19].Intriguingly,polymorphisms in the *Alox12* gene have been linked with early age natural menopause in various human populations [20, 21], implying that their expression may be key to follicular reserve later in life.Estrogen biosynthesis and proteolysis are both important processes involved in ovarian follicular development though they may be influenced by cytochrome P450 (CYP)-dependent fatty acid metabolites. However, CYP-dependent lipid metabolism has not been characterized with respect to follicular maturation in vivo. The dynamic changes in follicular CYP-dependent arachidonic acid metabolites and their modulatory function in vascular models suggest roles for these metabolites in follicular maturation. This may include regulation of estradiol biosynthesis and preovulatory remodeling of the follicular wall that should be fully explored in future studies [22]. Folliculogenesis is an ordered sequence of oocyte development and maturation;it involves the proliferation and differentiation of granulosa cells and is crucial for mammalian reproduction [23]. During follicular development, a series of changes occur. First, the primordial follicle grows into a growth follicle with somatic cells proliferation after which the follicular antrum is formed as the ovarian granulosa cell undergoes proliferation and differentiation. The majority of follicles then become atresia and only few grow dramatically to become dominant follicles and ovulation follows. Follicular fluid (FF) within the follicular antrum is the microenvironment of oocyte. It contains hormones, growth factors, proteins and phospholipids which are partially produced by Granulosa cells(GCs). Phospholipids are the most abundant lipids in the cell membrane and they include Lysophosphatidic Acid (LPA), LysophosphatidylCholine (LPC), Sphingosine-1 Phosphates and Sphingophoryl Choline [24]. Some studies have shown that LPC is metabolized into LPA by phospholipase [25, 26].

A potential biological mechanism by which LPC could influences mitochondrial oxidative capacity is through its role in the synthesis pathway of cardiolipin. Cardiolipin is a unique dimeric phospholipid containing four fatty acid chains that are specific to mitochondria. Cardiolipin is an essential constituent of mitochondrial membranes [27]. LPC in human plasma can be generated by three activities: phospholipase A2 (PLA2) on phosphatidylcholine, by the activity of endothelial lipase (EL) including phospholipase A1 (PLA1) on high-density lipoprotein [28] and from phosphatidylcholine during the formation of cholesteryl esters. LPC is also found in membranes both mitochondria and endoplasmic reticulum [29, 30]. Synthesis of cardiolipin involves LPA and phosphatidic acid (PA). LPC can be hydrolyzed to LPA by autotaxin, a secreted glycoprotein that is widely expressed in tissues [31, 32]. LPA can also be formed from the acylation of glycerol-3-phosphate on the outer mitochondrial membrane. Two isoforms of acylglycerol-3-phosphate acyltransferase (AGPAT); AGPAT 4 and 5 are located on the outer mitochondrial membrane and catalyze the acylation of LPA to PA [33]. PA is transferred to the inner mitochondrial membrane and is converted to nascent cardiolipin via CDP-glycerol, phosphatidylglycerophosphate, and phosphatidylglycerol. LPCs are highly mobile within intact cells and thus are good candidates for a cytoplasmic messenger that transduces signals to activate downstream processes and gene expression in the nucleus [34]. LPC can activate several second messengers; extracellular-signal-regulated kinases and protein kinase C, which have been shown to be involved in the regulation of follicular development and oocyte maturation [35]. In humans, blood triglyceride levels tend to increase, while blood lysophosphatidylcholine levels tend to decrease with age. Specific sphingolipid and phospholipid blood profiles have also been shown to change with age and are associated with exceptional human longevity. These data suggest that lipid-related interventions may improve human health span and that blood lipids likely represent a rich source of human aging biomarkers [36].

## Conclusions

Using advanced SWATH™-based mass spectrometry, we investigated metabolic changes in the follicular fluid of the younger and older patients.Metabonomic analysis of follicular fluid showed that several major metabolites changed with age, with some upregulated: arachidonic acid, LysoPC (16:1), LysoPC (20:4) and LysoPC (20:3), while others downregulated: LysoPC (18:3) and LysoPC (18:1). Among these metabolites, significant changes will take place in lipid metabolism, mainly characterized by the up-regulation of arachidonic acid, which affects oocyte development, while LPC participates in the regulation of follicular development and oocyte maturation, and its complex changes are closely related to follicular development. These differential metabolites related to follicular development may provide possible detection and therapeutic targets for promoting oocyte health. To provide scientific basis for understanding the environment of oocyte development.

## Data Availability

The datasets used and analyzed during the current study are available from the corresponding author on reasonable request.

## Abbreviations

SWATH: Sequential window acquisition of all theoretical fragment-ion spectra
MRM: Multiple reaction monitor
PCA: Principal component analysis
PLS-DA: Partial least squares discriminant analysis
FF: Human follicular fluids
AA: Arachidonate
LPC: Lysophosphatidylcholine
QC: Quality control
UPLC-Q-TOF: Ultra-performance liquid chromatography quadrupole time-of-flight mass spectrometry
CYP: Cytochrome P450
GCs: Granulosa cells
LPA: Lysophosphatidic Acid
PLA2: Phospholipase A2
EL: Endothelial lipase
PLA1: Phospholipase A1
PA: Phosphatidic acid
AGPAT: Acylglycerol-3-phosphate acyltransferase
